# LncRNA JPX promotes cervical cancer progression by modulating miR-25-3p/SOX4 axis

**DOI:** 10.1186/s12935-020-01486-3

**Published:** 2020-09-09

**Authors:** Xia Chen, Jingxiu Yang, Yuping Wang

**Affiliations:** Department of Gynaecology and Obstetrics, The Affiliated Lianyungang Oriental Hospital of Xuzhou Medical University, No. 57 Zhong’hua Rest Road, Lianyun District, Lianyungang, 222042 Jiangsu China

**Keywords:** JPX, miR-25-3p, SOX4, Cervical cancer

## Abstract

**Background:**

The long noncoding RNA (lncRNA) JPX is a molecular switch for X-chromosome inactivation. Accumulating studies have shown that the aberrant expression and function of lncRNAs are involved in the occurrence and development of tumors. However, the functional importance and mechanism of the action of lncRNA JPX in cervical cancer (CC) remain unknown.

**Method:**

In this study, qRT-PCR and western blotting were used to evaluate the mRNA or protein expression of JPX, miR-25-3p and SOX4 in CC tissues and cell lines. StarBase v2.0 database, luciferase reporter assay and RNA immunoprecipitation assay were used to explore the relationship between JPX and miR-25-3p. EdU assay, CCK-8 assay and transwell assay were utilized to evaluate the proliferation, migration and invasion of CC cells. The tumor xenograft assay in nude mice was performed to demonstrate the role of the JPX/miR-25-3p/SOX4 axis in CC.

**Results:**

We found that JPX was markedly upregulated, whereas miR-25-3p was markedly downregulated in CC tissues and cell lines, and the expression of JPX was negatively correlated with miR-25-3p in CC tissues. Moreover, overexpression of JPX increased proliferation, migration and invasion of HeLa cells, whereas knockdown of JPX decreased proliferation, migration and invasion of HeLa cells. In contrast to JPX, overexpression of miR-25-3p decreased proliferation, migration and invasion of HeLa cells. In addition, knockdown of JPX was found to inhibit HeLa cell viability and tumor development via up-regulating the expression of miR-25-3p and inhibiting the expression of SOX4.

**Conclusions:**

Our study demonstrates that JPX promotes cervical cancer progression through modulating the miR-25-3p/SOX4 axis, and may serve as a potential target for CC therapy.

## Background

Cervical cancer (CC) ranks the second in the world’s most common female malignancies and is still one of the leading causes of cancer-related deaths in women [1, 16]. Continuous infection of human papilloma virus is a prerequisite for CC precancerous lesions and CC [21]. Although it can be cured early with radical surgery, radiotherapy and chemotherapy, the prognosis of some patients with high risk factors is still not optimistic [29]. Therefore, it is urgent to investigate new therapeutic targets for the prevention and treatment of CC.

LncRNAs consist of more than 200 nucleotides, which are important members of non-coding RNAs and cannot be translated into proteins [19]. Studies have found that lncRNAs are a new type of tumor master regulators, which play key roles in important biological processes such as cell proliferation, epigenetic regulation, and transcription [22]. Accumulating evidence has shown that lncRNAs play a role in promoting or suppressing the occurrence and development of CC by regulating E6 and E7 oncoproteins in HPV, trapping lncRNA to regulate miRNA, and then Wnt and other signal pathways [7]. For example, lncRNA MEG3 was reported to be down-regulated in CC and affected cell proliferation and apoptosis by regulating miR-21 [32]. Long non-coding RNA C5orf66-AS1 promotes cell proliferation in cervical cancer by targeting miR-637/RING1 axis[20]. In addition, it has been reported that the aberrant expression of lncRNA JPX is related with the development of hepatocellular carcinoma [15] and lung cancer [8]. LncRNA JPX is a molecular switch for X chromosome inactivation [28]. As X chromosome inactivation is closely associated with CC, we speculated whether JPX is involved in the pathogenesis of CC [11]. In this study, we found that JPX was highly expressed in CC tissues and cell lines. The abnormal expression of JPX predicted that JPX might play an important role in CC.

MicroRNAs (miRNAs) are a class of small non-coding RNAs, which can be regulated by lncRNAs in human cancers [30]. It has reported that miR-25-3p affects the cisplatin-resistance CC cells [23]. However, the specific biological role of miR-25-3p is still not completely clarified in human CC. SOX4 has also been reported to contribute to the progression of CC and the resistance to the chemotherapeutic drug through ABCG2 [26], but the underlying mechanism remains unknown. In addition, bioinformatics analyses have found that miR-25-3p could bind with SOX-4. However, it has not been clarified how miR-25-3p and SOX-4 is involved in CC.

In this study, we found that miR-25-3p was the target of JPX based on bioinformatics analysis and luciferase reporter analysis. JPX plays a vital role in CC pathogenesis via modulating miR-25-3p/SOX4 axis. All these findings might help to investigate the pathogenesis and explore novel therapeutic targets for cervical cancer.

## Methods

### Tissue samples and cells

A total of 39 human cervical tissue samples and their corresponding adjacent non-carcinoma tissue samples were used in this study. All tissue samples were collected by the Affiliated Lianyungang Oriental Hospital of Xuzhou Medical University from Dec 2,017 to Apr 2,019 (Clinical pathological features of patients with cervical cancer were shown in Additional file 1: Table S1). Patients with primary cervical squamous cell carcinoma and complete clinical data were included. However, all patients who received radiotherapy or chemotherapy prior to surgery were excluded. The tissue specimens were immediately placed in liquid nitrogen after collection and transferred into a refrigerator at -80 ° C. This research had approval from the Ethics Committee of the Affiliated Lianyungang Oriental Hospital of Xuzhou Medical University (No. 312253LOHXMU). All registered patients have signed the written informed consent, and all relevant investigations have been conducted following Helsinki Declaration. Cells were incubated in DMEM medium (Thermo Fisher Scientific, USA) with 10% FBS and 0.5% penicillin/streptomycin (Gibco, USA) at 37 °C with 5% CO_2_.

### Plasmid construction and transfection

Plasmid preparation and transfection were carried out as previously described [9]. Hela cells were cultured in minimum essential medium (MEM; Thermo fisher, USA), supplemented with 10% fetal bovine serum, and incubated under 5% CO_2_ at 37 °C. For transfection, HeLa cells were seeded in six-well plates (2 × 10^5^ cells per well), cultured overnight at 37 °C in 5% CO_2_ and grown to 70% confluence prior to transfection. Transfection with pc-NC, pc-JPX, sh-NC, sh-JPX, miR-NC or miR-25-3p mimic was performed by Lipofectamine 2000 following the manufacturer’s instructions. pc-NC, pc-JPX, sh-NC, sh-JPX, miR-NC and miR-25-3p mimic were purchased from GenePharma (Shanghai, China). The sequences of the sh-NC and sh-JPX were as follows:

**Table:** 

sh-NC	Sense: 5’- UUCUCCGAACGUGUCACGUTT − 3′ Anti-sense: 5’- ACGUGACACGUUCGGAGAATT − 3′
sh-JPX miRNA-NC miR-25-3p mimic	Sense: 5′-CCAGUUAAUAGUAUUGUGUTT-3′ Anti-sense: 5′-ACACAAUACUAUUAACUGGT − 3′ Sense: 5′-UUCUCCGAACGUGUCACGUTT-3′ Anti-sense: 5′-ACGUGACACGUUCGGAGAATT-3′ Sense: 5′-CAUUGCACUUGUCUCGGUCUGA-3′ Anti-sense: 5′-AGACCGAGACAAGUGCAAUGUU-3′

### EdU assay

EdU assay was carried out to determine cell proliferation as previously described [27]. Cells in logarithmic growth phase were seeded into a 96-well plate with a cell density of 4 × 10^3^ cells per well. Next, 100 ml of 50 mM EdU culture medium was added into each well and incubated for 2 h. After that, cells were incubated with 50 ml of stationary liquid (PBS containing 4% polyoxymethylene) at room temperature for 30 min, followed by incubation with 50 ml of 2-mg/ml glycine for 5 min and permeabilized with 100 ml of penetrating agent (PBS containing 0.5% TritonX-100) for 10 min. After being washed with PBS, the cells were treated with 100 ml of 13 Apollo staining reaction liquid (Guangzhou RiboBio, Guangzhou, Guangdong, China) for 30 min followed by the addition of 100 ml of penetrating agent and 100 ml of methanol for washing. Cells were subsequently washed with PBS and added with 100 ml of 13 Hoechst 33,342 reaction reagents for 30 min. Apollo and 4′, 6-dimidyl-2-phenylindole were screened to record EdU-positive cells by fluorescent microscope.

### CCK-8 assay

CCK-8 assay was carried out to determine cell viability as previously described [13]. Cells were respectively seeded into 96-well plates at a density of 5 × 10^3^ cells/well, with each experiment being repeated five times. After being incubated at 37 °C for 24 h, 20 µl CCK-8 reagent was added into each well for another 1 h of incubation at 37 °C following the manufacturer’s instructions (Beyotime, China). Next, optical density (OD) values were measured at 450 nm using a microplate reader (Thermo, USA).

### Transwell assay

Transwell assay was carried out as previously described [14]. Cells (3 × 10^5^) were plated onto a Matrigel-coated membrane in the upper chamber of a 24-well insert with the serum-free medium. Culture medium containing 10% fetal bovine serum was added into the basolateral chamber and left to incubate at 37 °C for 24 h. The transwell chamber was washed by PBS twice 5 min each time, fixed with 5% pentanediol at 4 °C, stained by 0.1% crystal violet for 30 min, washed with PBS twice, and observed under a microscope. The numbers of cells that passed through the Matrigel were used as the index to evaluate how well the cells were able to invade. Assays were performed in duplicate in three independent experiments.

#### RNA extraction and quantitative real-time PCR (qRT- PCR)

RNA extraction and quantitative real-time PCR (qRT-PCR) were carried out as previously described [9]. Total RNAs were extracted using Trizol reagent (Invitrogen, Carlsbad, CA). All reagents for qRT-PCR were purchased from Takara (Dalian, China). For mRNA analyses, the first-strand cDNA was generated from RNA (500 ng) using the PrimeScript® RT reagent Kit following the manufacturer’s instructions. Prime Script™ RT master mix was used for reverse transcription of RNA, and SYBR Premix Ex Taq II (TaKaRa Biotechnology Company, Dalian, China) and 10 pg cDNA were used for quantitative PCR (qPCR). The polymerase chain reaction was performed with ABI Prism 7900HT (Applied Biosystems, USA), which was set up to run the following program: 95 °C for 10 min, 95 °C for 30 s, and 60 °C for 30 s, followed by 38 cycles of 74 °C for 25 s. qPCR data for mRNAs were normalized to GAPDH, and qPCR data for miR-145-5p were normalized to U6. Relative quantitation was calculated using the 2^−ΔΔCT^ method [12]. The primers used in this study were:

**Table:** 

GAPDH	F: AAGAAGGTGGTGAAGCAGGC R: GTCAAAGGTGGAGGAGTGGG
U6 SOX4 JPX miR-25-3p	F: ATTGGAACGATACAGAGAAGATT R:GGAACGCTTCACGAATTTG F: GTGAGCGAGATGATCTCGGG R:CAGGTTGGAGATGCTGGACTC F: TGCAGTCAGAAGGGAGCAAT R: CACCGTCATCAGGCTGTCTT F: CATTGCACTTGTCTCGGTCTGA R: GCTGTCAACGATACGCTACGTAACG

## Luciferase reporter assay

Luciferase reporter assay was carried out as previously described [28]. A hypothetical target for miR-25-3p was predicted using miRDB and microRNA. Lipofectamine 2,000 (Invitrogen) was used to transfect EGFP reporter plasmid with SOX4 3′-UTR or SOX4 3′-UTR mut and miR-25-3p mimic or miR-25-3p inhibitor into HeLa cells, a plasmid expressing red fluorescent protein (RFP) was a control. Cells were lysed at 2 d after transfection and the intensity of EGFP and RFP fluorescence was measured. JPX’s hypothetical ceRNA was predicted by Starbase 3.0. pGL3-JPX or pGL3-JPX mut had transfection to HeLa cells. pRL-TK vector was co-transfected as a control. At specified time points, the luciferase assay was performed (Promega, USA).

## Tumor xenograft assay

The tumor xenograft assay was carried out as previously described [17]. A total of 15 six-week-old male BALB/c athymic nude mice (20–30 g) were provided by Chinese Academy of Sciences, Shanghai. They were divided into 3 groups and maintained at 24 °C with 50% humidity. They were free to eat or drink with half day in light and half day in dark. To establish the xenograft model, the transfected Hela cells were purified with 5 µg/ml puromycin to obtain cells with stable JPX knockdown by transfecting shJPX or shJPX + miR-25-5p inhibitor. Then, 3 × 10^6^ these purified Hela cells with Ctrl, shJPX or shJPX + miR-25-5p inhibitor were subcutaneously inoculated into the mice. HeLa cells were administered subcutaneously to the right side near the forelimbs. The tumor growth was tested every week for 4 weeks. Tumor volume was calculated as length (mm) × width^2^ (mm^2^)/2. At 4 weeks after transfection, mice were sacrificed and the tumors were sectioned, weighed and embedded with paraffin. Then, the sample was de-paraffinized. Heat-induced antigen retrieval was performed. Next, the sample was treated with a primary antibody against Ki-67 (1:500; Abcam, UK), then 5% goat serum and 3% hydrogen peroxide. The immunohistochemistry results were recorded by a microscope. The proliferation index was determined by Ki67 immunostaining and calculating the ratio of Ki67-positive cells among the total number of cells in five randomly selected fields. All animal experimental protocols were approved by the Laboratory Animal Care and Use Committee of The Affiliated Lianyungang Oriental Hospital of Xuzhou Medical University.

## RNA immunoprecipitation (RIP) assay

RIP assay was carried out as previously described [20] using the RIP kit (Millipore, USA). Cells had lysis and treatment with magnetic beads conjugated to human anti-Ago2 antibodies or control antibody (Millipore, USA).

## Western blotting

Western blotting was carried out as previously described [13]. HeLa cells were lysed. Proteins were separated by 10% SDS-PAGE and sent to Millipore membranes, USA. Prior to treatments with anti-SOX4 or anti-GAPDH (1:1000; Abcam, UK), the membrane was blocked by 5% skim milk in Tris buffered saline containing Tween-20 at room temperature for approximately 2 h. The antibodies were incubated at 4 °C overnight. Subsequently, the membranes were conjugated with an HRP antibody (1:3000; CST, USA) at 37 °C for 1 h. ECL was employed to measure immune response bands. Image Pro Plus software v.6.0 (Media Cybernetics, Inc., Rockville, MD, USA) was used to scan the gray value of target protein and internal parameter, and the ratio of the gray value of the target protein to the gray value of the GAPDH was used to characterize the content of the target protein. The larger the ratio, the greater the target protein content.

### Statistical analysis

All the data were collected and expressed as the Mean ± Standard Deviation (SD) from the experiments repeated at least three times and analyzed using GraphPad. The Student’s t-test was used to compare two groups. One-way ANOVA was used for analyzing the statistical significances of multiple groups. The Kaplan-Meier method was employed to generate survival curves and log-rank test was used to analyze the differences. *p* < 0.05 was considered statistically significant.

## Results

### The expression of JPX and miR-25-3p in CC tissues and cell lines

**Fig. 1:**
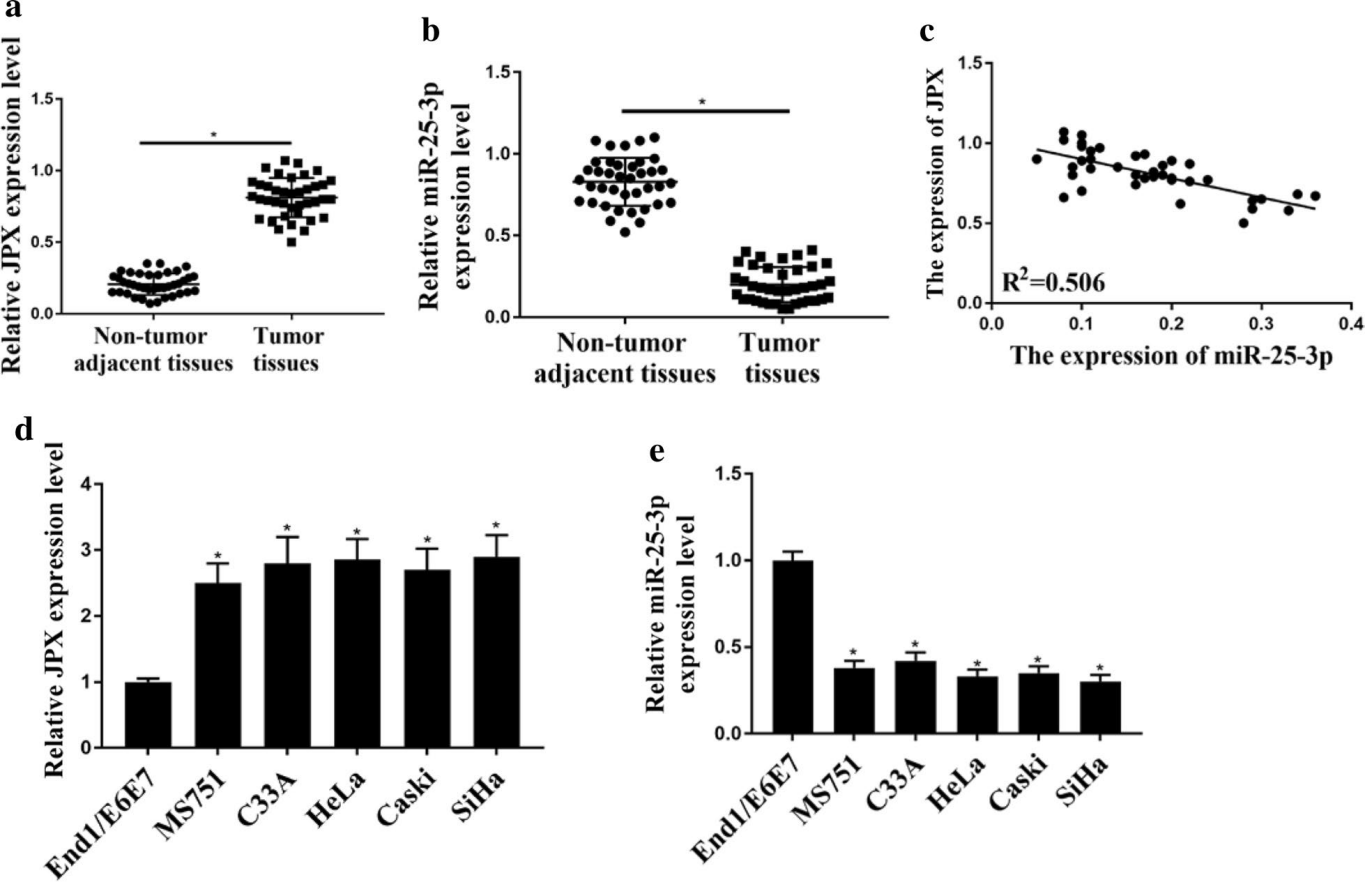
The expression of JPX and miR-25-3p in CC tissue and cell lines. **a**, **b** The expression of JPX and miR-25-3p in normal adjacent tissue samples (n = 39) and CC tissue samples (n = 39) detected by qRT-PCR. **c** The relationship of JPX and miR-25-3p in CC tissue samples analyzed in starBase v2.0 database. **d**, **e** The expression of JPX and miR‑25‑3p in CC cell lines (MS751, C33A, HeLa, Caski, and SiHa) and normal cervical cells (End/E6E7) was determined. *p < 0.001, n = 03

QRT-PCR was used to evaluate the expression of JPX and miR-25-3p in CC tissues and cell lines. As shown in Fig. 1, the expression levels of JPX were markedly increased in CC tissue samples in comparison to that in normal adjacent tissue samples (Fig. 1a, p < 0.05), whereas the expression levels of miR-25-3p in CC tissue samples were markedly decreased than that in normal adjacent tissue samples (Fig. 1b, p < 0.05). Furthermore, using starBase v2.0 database to explore the relationship between JPX and miR-25-3p based on their qRT-PCR data, we found that the expression of JPX was negatively correlated with the expression of miR-25-3p (Fig. 1c). In addition, the expression of JPX was markedly elevated in CC cell lines compared to that in End/E6E7 cells (Fig. 1d, p < 0.05), whereas the expression levels of miR-25-3p were markedly decreased in CC cell lines in comparison to End/E6E7 cells (Fig. 1e, p < 0.05). These results suggest that the expression of JPX and miR-25-3p is abnormal in cervical cancer, and there may be a correlation between the expression of JPX and miR-25-3p.

## The function of JPX in CC cells lines

**Fig. 2:**
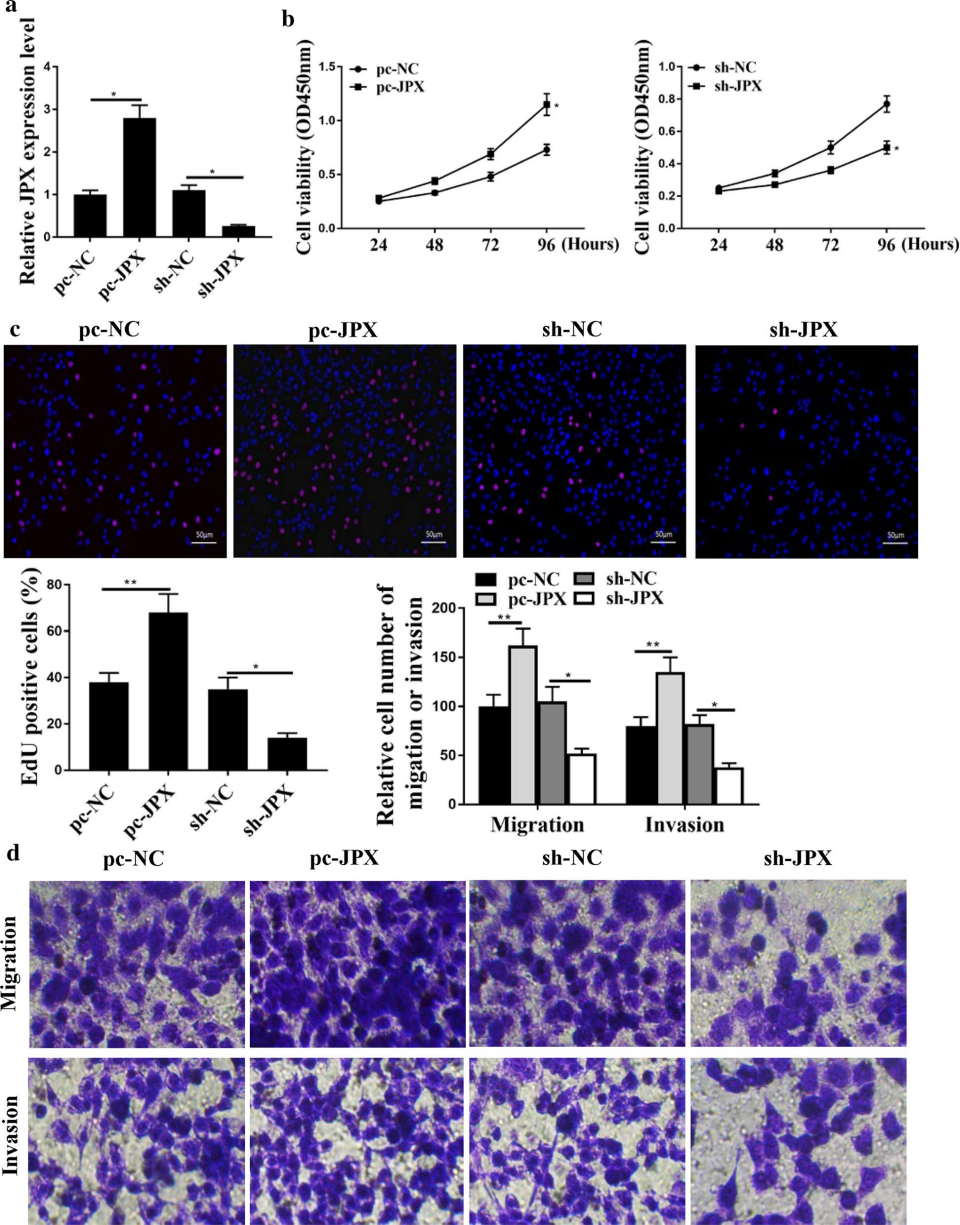
The function of JPX in CC cells lines. **a** The expression of JPX in HeLa cells transfected with pc-NC, pc-JPX, sh-NC, or sh-JPX. **b** CCK8 assay for viability of HeLa cells transfected with pc-NC, pc-JPX, sh-NC, or sh-JPX. **c** EdU assay for proliferation abilities of HeLa cells transfected with pc-NC, pc-JPX, sh-NC, or sh-JPX. **d** Transwell assay for migration and invasion abilities of HeLa cells transfected with pc-NC, pc-JPX, sh-NC, or sh-JPX. *p < 0.01, **p < 0.05. n = 0.03

To investigate the roles of JPX play in CC, we transfected HeLa cells with pc-NC, pc-JPX, sh-NC, or sh-JPX. As shown in Fig. 2a, the expression of JPX in HeLa cells was successfully upregulated (*p* < 0.05) and downregulated (*p* < 0.05). Moreover, upregulated expression of JPX markedly increased cell proliferation of HeLa cells (Fig. 2b, c, *p* < 0.05), whereas downregulated expression of JPX decreased cell proliferation of HeLa cells (Fig. 2b, c, *p* < 0.05). In addition, upregulated expression of JPX markedly increased cell migration (*p* < 0.01) and invasion (*p* < 0.05) of HeLa cells, whereas downregulated expression of JPX decreased cell migration (*p* < 0.01) and invasion (*p* < 0.05) of HeLa cells (Fig. 2d). These results suggest that lncRNA JPX plays an important role in proliferation, migration and invasion of CC cells.

## The function of miR-25-3p in CC cell lines

**Fig. 3:**
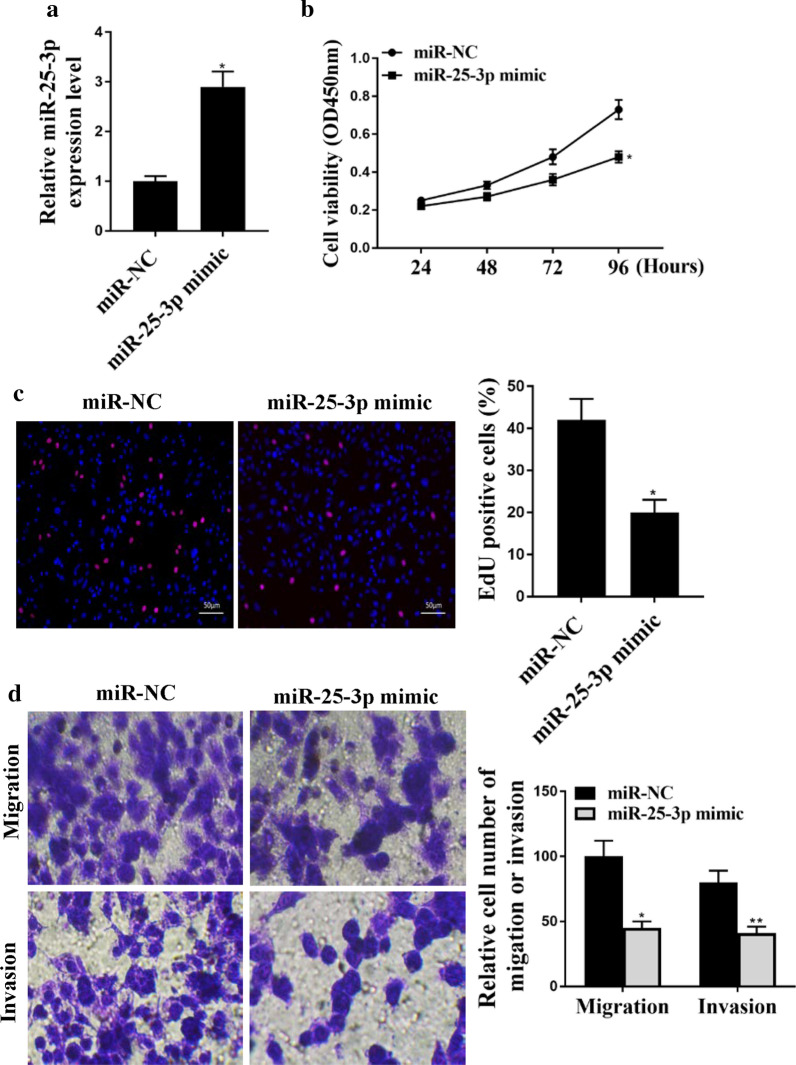
The function of MiR-25-3p in CC cell lines. **a** The expression of miR-25-3p in HeLa cells transfected with miR-NC or miR-25-3p mimic. **b** CCK8 assay for viability of HeLa cells transfected with miR-NC or miR-25-3p mimic. **c** EdU assay for cell proliferation abilities of HeLa cells transfected with miR-NC or miR-25-3p mimic. **d** Transwell assay for migration and invasion abilities in HeLa cells transfected with miR-NC or miR-25-3p mimic. *p < 0.01, **p < 0.05. n = 3

To investigate the roles of miR-25-3p play in CC, we transfected HeLa cells with miR-NC and miR-25-3p mimic. As shown in Fig. 3a, the expression of miR-25-3p was upregulated by transfected with miR-25-3p mimic compared with that transfected with miR-NC (Fig. 3a, p < 0.05). Moreover, upregulated expression of miR-25-3p markedly decreased cell proliferation of HeLa cells (Fig. 3b, c and p < 0.05). Meanwhile, the migration and invasion of HeLa cells also significantly decreased after upregulated expression of miR-25-3p (Fig. 3d, migration: *p* < 0.05, invasion: *p* < 0.01). Taken together, these results suggest that the upregulation of miR-25-3p inhibit the proliferation, migration and invasion of CC cells.

## The relationship between JPX and miR‑25-3p

**Fig. 4:**
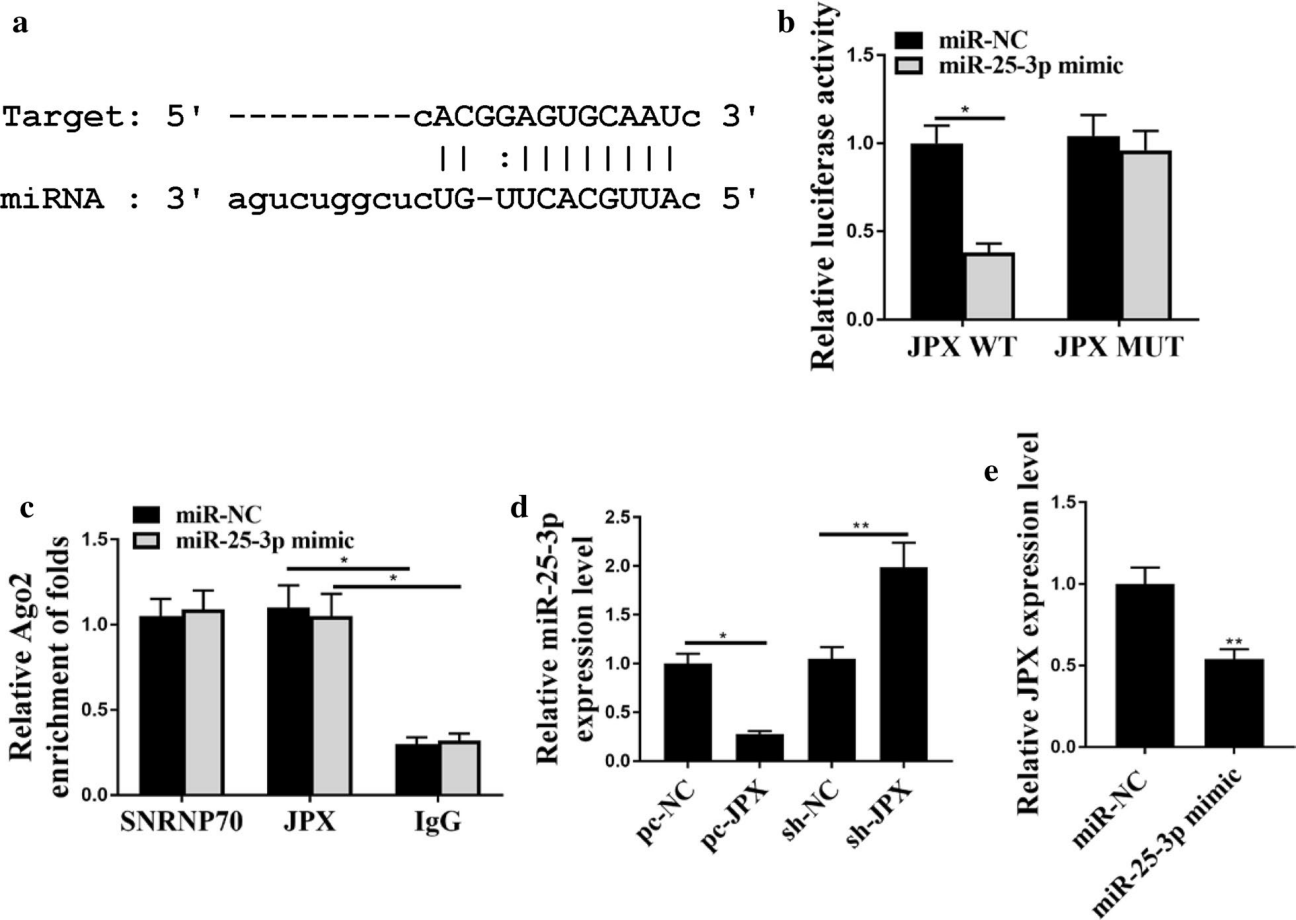
The relationship between JPX and miR‑25-3p. **a** The common binding sites between JPX and miR‑25-3p. **b **The interactions between JPX and miR‑25-3p were evaluated by luciferase assay. **c** RIP assay. **d** miR-25-3p levels in HeLa cells. **e** JPX levels in HeLa cells. *p < 0.01. n = 03

As shown in Fig. 4a, JPX had common binding sites with miR-25-3p, suggesting that miR-25-3p might bind with JPX. Luciferase assay showed that miR-25-3p mimics markedly decreased the luciferase activities of WT‑JPX (*p* < 0.05), but not MUT-JPX luciferase activities (Fig. 4b). Furthermore, RIP assay also showed that WT-miR-25-3p bound more to JPX than Mut-miR-25-3p (Fig. 4c, p < 0.05). These results suggest that miR-25-3p may directly bind to JPX. To further investigate the correlation between JPX and miR-25-3p, HeLa cells were transfected with pc-NC, pc-JPX, sh-NC, or sh-JPX, and the results showed that upregulated expression of JPX markedly decreased the expression levels of miR-25-3p (*p* < 0.05), whereas downregulated expression of JPX markedly increased the expression levels of miR-25-3p (*p* < 0.01) (Fig. 4d). In addition, upregulation of miR-25-3p markedly decreased the expression levels of JPX in HeLa cells (Fig. 4e, p < 0.01). Taken together, these results suggest that the expression of miR-25-3p can be regulated by JPX, and miR-25-3p may be the downstream target of JPX signaling pathway.

### MiR-25-3p mediated the function of JPX on cell progression in CC cells

**Fig. 5:**
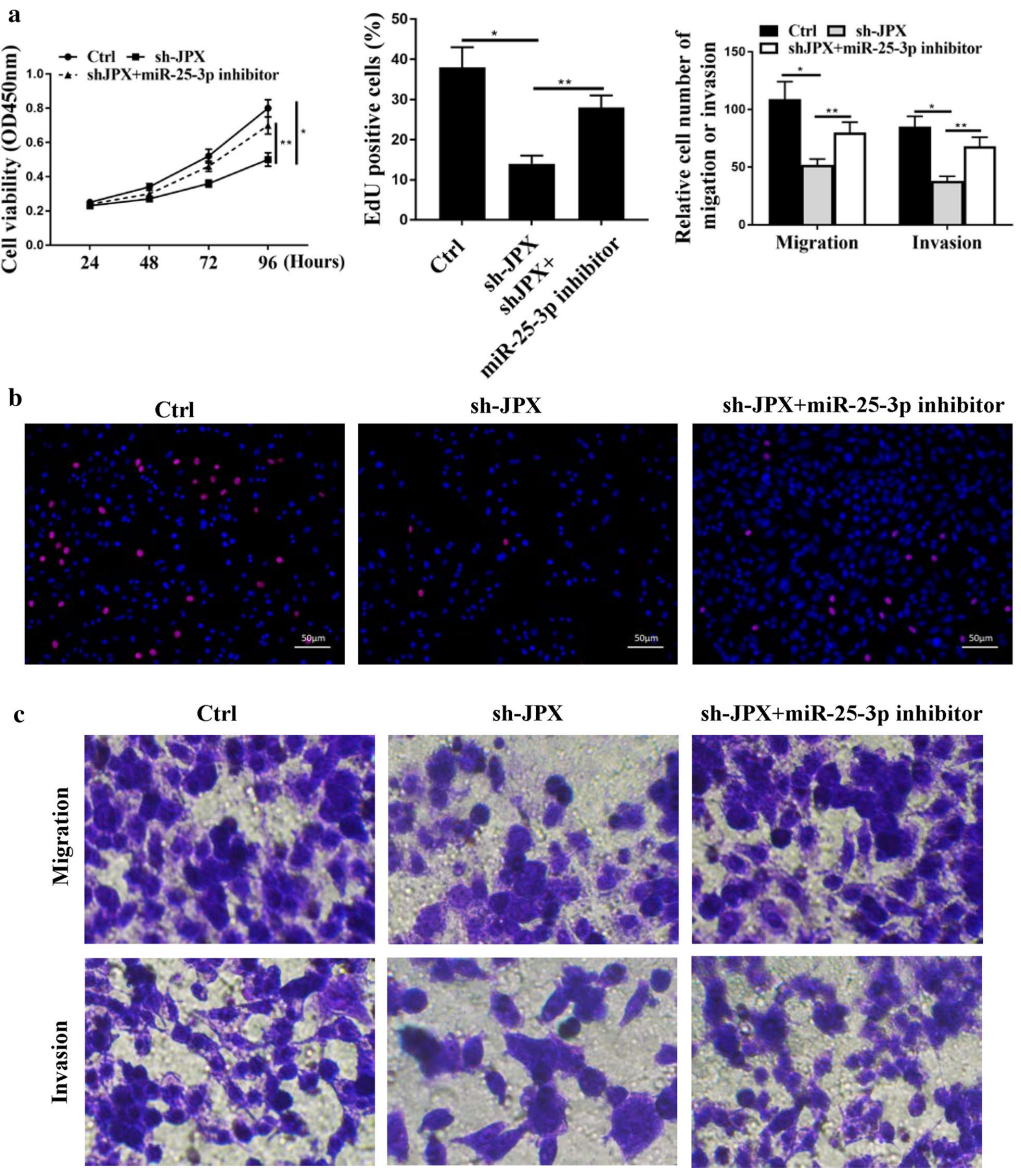
MiR-25-3p mediated the function of JPX on cell progression in CC cells. **a** CCK8 assay for cell viability of HeLa cells transfected with control, sh-JPX, or shJPX + miR-25-3p inhibitor. **b** EdU assay for cell proliferation of HeLa cells transfected with control, sh-JPX, or shJPX + miR-25-3p inhibitor. **c** Transwell assay for migration and invasion abilities of HeLa cells transfected with control, sh-JPX, or shJPX + miR-25-3p inhibitor. *p < 0.01, **p < 0.05. n = 03

Since the expression of miR-25-3p can be regulated by JPX, whether miR-25-3p can mediate the role of JPX play in CC cells was then evaluated. As shown in Fig. 5a and B, downregulated expression of JPX markedly decreased proliferation of HeLa cells (*p* < 0.05), and that was attenuated by miR-25-3p inhibitor (*p* < 0.01). In addition, downregulated expression of JPX also markedly decreased migration and invasion of HeLa cells (migration: *p* < 0.05, invasion: *p* < 0.05), and that was also attenuated by miR-25-3p inhibitor (Fig. 5c, migration: *p* < 0.01, invasion: *p* < 0.01). Therefore, these results reveal that miR-25-3p certainly mediate the roles of JPX in cell progression of CC cells.

## The relationship between miR-25-3p and SOX4

**Fig. 6:**
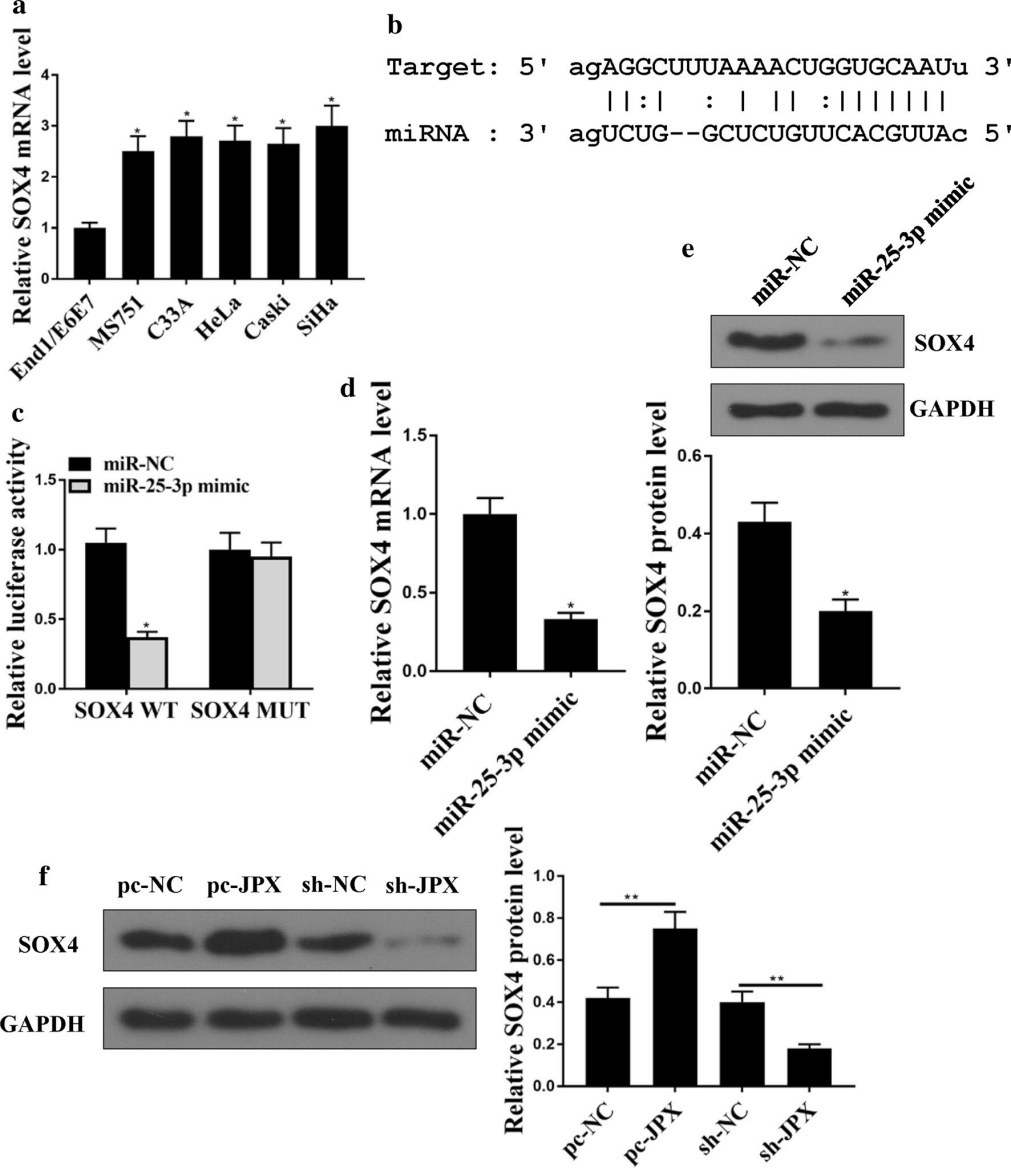
The relationship between MiR-25-3p and SOX4. **a** The expression of SOX4 in CC cell lines (MS751 C33A HeLa Caski SiHa) and normal cervical cells (End/E6E7) was detected. **b** The common binding sites between SOX4 and miR‑25-3p.**c** The interactions between SOX4 and miR‑25-3p was evaluated by luciferase assay. **d**, **e** SOX4 protein and mRNA expression in HeLa cells were evaluated by western blotting analysis and RT-PCR. **f** Western blot analysis of SOX4 protein expression in HeLa cells transfected with pc-NC, pc-JPX, sh-NC, or sh-JPX. *p < 0.01, **p < 0.05. n = 03

It has been reported that SOX4 contributes to the progression of cervical cancer[25]. So, we evaluated that the expression of SOX4 in CC cell lines. As shown in Fig. 6a, the expression levels of SOX4 were increased in CC cell lines (Fig. 6a, p < 0.05). Moreover, bioinformatics analysis showed a common binding sites with miR-25-3p in SOX4 (Fig. 6b), suggesting that miR-25-3p might bind with SOX4. Luciferase assay confirmed that miR-25-3p mimics markedly decreased the luciferase activity of WT-SOX4 (*p* < 0.05), but not MUT-SOX4 luciferase activities (Fig. 6c). Furthermore, the expression levels of SOX4 were reduced after miR-25-3p mimic was transfected in HeLa cells at both mRNA and protein levels (Fig. 6d, mRNA: *p* < 0.05; and Fig. 6e, protein: *p* < 0.05). In addition, downregulation of JPX markedly decreased the expression levels of SOX4 in HeLa cells (Fig. 6f, mRNA: *p* < 0.01, protein: *p* < 0.01). These results illustrate that SOX4 was targeted by miR-25-3p in CC cells.

### The role and underlying molecular mechanism of JPX in tumorigenesis of CC

**Fig. 7:**
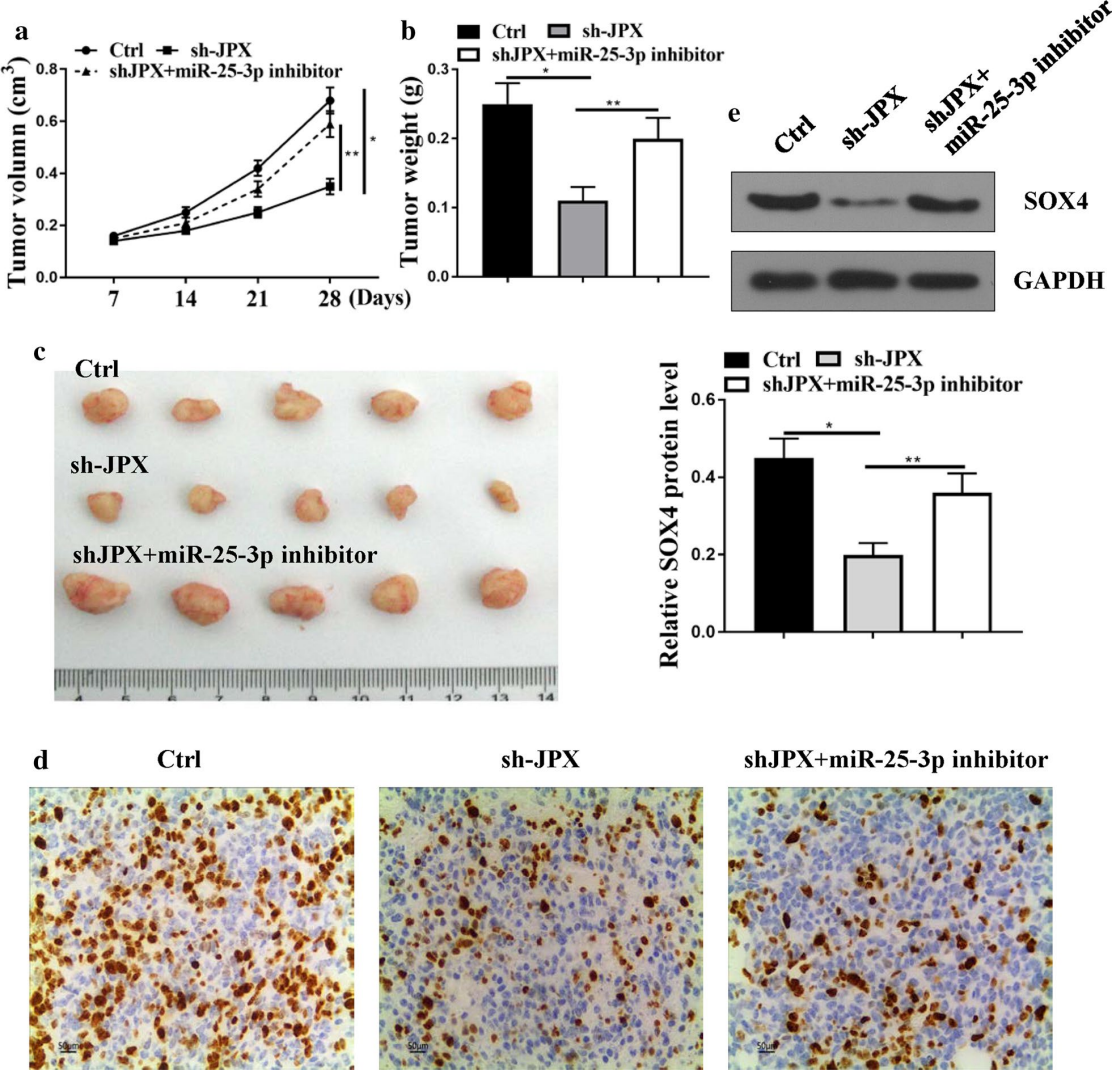
The role and underlying molecular mechanism of JPX in tumorigenesis of CC. **a** Tumor volumes were measured every 1 week, and growth curves were shown. **b** Tumor weight. **c** Representative images of mice bearing tumors. **d** representative images for Ki67 immunostaining of tumor samples from different groups. **e** Western blot analysis of SOX4 in tumor tissue samples. *p < 0.01, **p < 0.05. n = 5

The tumor xenograft assay in nude mice was conducted to evaluate the role of JPX in the tumorigenesis of CC. The tumor growth curve and tumor volume were measured and recorded in every 7 days. We found that tumor growth curve and tumor volume decreased in the sh-JPX group (tumor growth curve: *p* < 0.05, tumor volume: *p* < 0.05), but not in sh-JPX and miR-25-3p inhibitor group (Fig. 7a, c). Moreover, Ki-67 staining assay showed that sh-JPX reduced proliferation in tumor tissue samples of mice, but miR-25-3p inhibitor could attenuate this effect (Fig. 7d). In addition, western blotting showed that the expression levels of SOX4 decreased in the sh-JPX group (*p* < 0.05), but miR-25-3p inhibitor attenuated this effect (Fig. 7e, p < 0.01). Taken together, these results suggest that JPX/miR-25-3p/SOX4 axis play an important role in tumorigenesis of CC.

## Discussion

Despite encouraging progress in understanding the molecular mechanism of CC development, the prognosis of some patients with high risk factors is still not optimistic [19]. Accumulative evidence has suggested that lncRNAs could act as molecular indicators for early diagnosis and prognostic evaluation [33], which are closely related to the occurrence and development of cervical squamous cell carcinoma, and are involved in regulating the molecular mechanism of cervical squamous cell carcinoma and the expression of estrogen and progesterone [19]. For instance, lncRNA CCHE1 was reported to promote cervical cancer cell proliferation via upregulating PCNA [31]. LncRNA XLOC_006390 facilitates cervical cancer tumorigenesis and metastasis as a ceRNA against miR-331-3p and miR-338-3p[13]. In the present study, lncRNA-JPX was found to be highly expressed in CC tissues and cell lines, and associated with larger tumor size and tumor cell proliferation. Moreover, JPX promoted proliferation, migration and invasion of CC cells by down-regulating miR-25-3p in a ceRNA mechanism via targeting on SOX4.

LncRNA JPX is an activator of XIST and a molecular switch for X-chromosome inactivation, and it has been reported to positively regulate the expression of XIST and therefore participate in X-chromosome inactivation [2, 24, 28]. Aberrant expression of XIST has been implicated to regulate cell migration and tumor metastasis in different kinds of cancer, highlighting its oncogenic role in cancer progression [3, 4, 30]. Although JPX is confirmed to involve in lung cancer, colorectal, hepatocellular and ovarian cancer, the expression, functional importance and mechanism of action of lncRNA JPX in CC remain unknown[23]. Here, for the first time, our study showed that the expression levels of JPX were markedly increased in CC tissue samples and cell lines in comparison to that in normal tissue samples and cells, consistent with the report that JPX was increased in non-small-cell lung cancer [8]. Furthermore, we found that JPX plays important roles in proliferation, migration and invasion of CC cells. Upregulated expression of JPX markedly increased proliferation, migration and invasion of HeLa cells, whereas downregulated expression of JPX decreased proliferation, migration and invasion of HeLa cells. Moreover, downregulated expression of JPX decreased tumor size and tumor cell proliferation. These findings suggest that JPX play an oncogenic role in CC, and may be a useful prognostic predictor of CC.

In recent years, ceRNAs have been reported to be a very important class of post-transcriptional regulators that affect the occurrence and development of tumor by altering the corresponding gene expression through miRNA-mediated mechanism [10]. As a type of ceRNA, lncRNAs can act as molecular sponges to adsorb miRNAs through the same miRNA response elements (MREs), thereby regulating their target genes and ultimately affecting tumor progression. For example, lncRNA SNHG20 promoted cell proliferation and invasion via miR-140-5p-ADAM10 axis in cervical cancer [6]. LncRNA HOXD-AS1 bound with miR-130a to downregulted the repression of E2F8, therefore regulating glioma development [5]. In this study, we found that the expression levels of miR-25-3p in CC tissue samples and cell lines were markedly lower than that in normal tissue samples and cells. Furthermore, combining lncRNA microarray and bioinformatic prediction, we also found that there were binding sites with miR-25-3p in JPX, and the expression of JPX was negatively correlated with miR-25-3p in cervical cancer tissues. Moreover, upregulated expression of miR-25-3p markedly decreased proliferation, migration and invasion of HeLa cells. Importantly, our results showed that downregulated expression of JPX markedly decreased proliferation, migration and invasion of HeLa cells, and that was attenuated by miR-25-3p inhibitor, suggesting that miR-25-3p mediate the roles of JPX in the progression of CC cells. These data altogether indicated that miR-145-5p is a crucial mediator for JPX to function as a ceRNA in CC.

It has been reported that SOX4 contributes to the progression of cervical cancer and the resistance to the chemotherapeutic drug through ABCG2 [26]. In consistence to this research, we also noticed that the expression levels of SOX4 were increased in CC cell lines. The bioinformatic analysis and Dual-Luciferase reporter assay showed that miR-25-3p directly inhibited the expression of SOX4. Furthermore, western blotting results showed that the expression of JPX was positively correlated with SOX4 in CC cells. In addition, in vivo assay confirmed that JPX promoted tumor growth, which was attenuated by adding miR-25-3p inhibitor. Taken together, these results demonstrate that JPX plays a critical role in CC pathogenesis and the JPX participates in the occurrence and development of CC through JPX/miR-25-3p/SOX4 axis. Although our study provides the first insights into the function and molecular mechanism of JPX in CC, the reason for the altered expression of JPX and the precise molecular mechanism underlying the action of JPX in CC ramian unknown. It has been reported that lncRNA JPX/miR-33a-5p/Twist1 axis regulates tumorigenesis and metastasis of lung cancer by activating Wnt/β-catenin signaling [18]. The signaling pathways involve in the molecular mechanism underlying the JPX/miR-25-3p/SOX4 axis in CC need to be investigated in the future work.

## Conclusions

Our results demonstrated that JPX played an important role in regulating proliferation, migration and invasion of CC tissues and cell lines via JPX/miR-25-3p/SOX4 axis, which provides new insights into prognostic diagnosis and therapeutic strategies for patients with CC.

## Supplementary information

**Additional file 1: Table S1. **Clinical correlation of JPX expression in CC (n = 39).

## Abbreviations

CC: Cervical cancer
lncRNA: Long non-coding RNA
EdU: 5-ethynyl-2′-deoxyuridine
RFP: Red fluorescent protein

## Availability of data and materials

The datasets used and/or analyzed during the current study are available from the corresponding author on reasonable request.
