# Supplementary material for: LncRNA JPX promotes cervical cancer progression by modulating miR-25-3p/SOX4 axis
Source: Cancer Cell Int. 2020 Sep 9;20:441. doi: 10.1186/s12935-020-01486-3 (PMC7487936; doi:10.1186/s12935-020-01486-3)
Supplement: Supplementary file 1 — Additional file 1: Table S1. Clinical correlation of JPX expression in CC (n = 39). [file 12935_2020_1486_MOESM1_ESM.docx]

**Additional file 1. Clinical correlation of JPX expression in CC (*n* = 39)**

| **Clinical parameters** | **Cases (n)** | **Expression level** | | ***P* value (* *p* < 0.05)** |
| --- | --- | --- | --- | --- |
|  |  | **JPX^high^(*n* = 22)** | **JPX^low^(*n* = 17)** |  |
| Age(years) | | | | |
| < 45 years | 16 | 9 | 7 | 0.009* |
| ≥ 45 years | 23 | 13 | 10 |  |
| FIGO stage | | | | |
| I | 25 | 10 | 15 | 0.005* |
| II | 14 | 12 | 2 |  |
| Tumor size (cm) |  |  |  | 0.002* |
| <4 | 20 | 11 | 9 |  |
| ≥ 4 | 19 | 11 | 8 |  |
| LNM |  |  |  | 0.04* |
| Negative | 24 | 13 | 11 |  |
| Positive | 15 | 9 | 6 |  |
| Vaginal invasion |  |  |  | 0.04* |
| Negative | 26 | 13 | 13 |  |
| Positive | 13 | 9 | 4 |  |
| Histology |  |  |  | 0.866 |
| Squamous | 29 | 17 | 12 |  |
| Adenocarcinoma | 10 | 5 | 5 |  |
| Parametrial extention |  |  |  | 0.433 |
| Negative | 30 | 15 | 15 |  |
| Positive | 9 | 7 | 2 |  |

*FIGO*: International Federation of Gynecology and Obstetrics; *LNM:* lymph node metastasis

*Statistically significant by Pearson chi-square test
